# Dual triggering fails to improve mature oocyte yield in poor responders: a cycle-based analysis in POSEIDON group 4 patients

**DOI:** 10.3389/fendo.2026.1870397

**Published:** 2026-09-24

**Authors:** Antonio Mercorio, Alexia Chatziparasidou, Nicholas Christoforidis, Achilleas Papatheodorou, Jean-Marc Ayoubi, Panagiotis Drakopoulos, Nicola Pluchino

**Affiliations:** 1 Assisted Reproductive Technology Unit, Department of Gynecology, Hôpital Foch, Suresnes, France; 2 Embryolab Fertility Clinic and Embryolab Academy, Thessaloniki, Greece; 3 In Vitro Fertilization (IVF) Unit, Institute of Life, Athens, Greece; 4 Faculty of Medicine, European University Cyprus, Nicosia, Cyprus; 5 Centre for Reproductive Medicine, Universitair Ziekenhuis Brussel, Vrije Universiteit Brussel, Brussels, Belgium; 6 Division of Gynaecology, Reproductive Medicine Unit, Geneva University Hospitals and Faculty of Medicine, University of Geneva, Geneva, Switzerland

**Keywords:** dual trigger, GnRH agonist, *in vitro* fertilization, oocyte maturation, poor ovarian response, POSEIDON classification

## Abstract

**Background:**

Poor ovarian responders (PORs) represent a challenging population in assisted reproductive technology, where even small increases in oocyte yield may meaningfully impact reproductive outcomes. Final oocyte maturation triggering is a critical step in controlled ovarian stimulation. While human chorionic gonadotropin (hCG) has traditionally been used alone, dual triggering—combining hCG with a gonadotropin-releasing hormone agonist (GnRHa)—has emerged as a strategy to enhance oocyte yield and maturation. However, evidence in PORs remains limited and inconsistent, likely reflecting the heterogeneity of the populations studied. Within the POSEIDON classification, group 4 patients—older women with reduced ovarian reserve—represent a relevant target for optimized stimulation strategies.

**Methods:**

This retrospective cohort study included GnRH antagonist IVF cycles in POSEIDON group 4 women (age ≥35 years with AFC <5 and/or AMH <1.2 ng/mL). Final oocyte maturation was triggered with recombinant hCG alone or dual trigger (GnRHa + hCG). The primary outcome was the number of mature (MII) oocytes per cycle. Secondary outcomes included total oocytes retrieved, fertilization rate, embryo utilization, and clinical outcomes. Analyses were performed using generalized estimating equations (GEE) to account for repeated cycles.

**Results:**

A total of 1,824 cycles were included (354 dual trigger, 1,470 hCG alone). No significant differences were observed in mature oocytes, maturation rate, or total oocytes retrieved between groups. Fertilization rate was slightly higher with dual trigger (74.1% vs. 69.9%; p = 0.04), whereas embryo utilization was lower (62.4% vs. 70.4%; p < 0.001). Clinical pregnancy and live birth rates were similar. In multivariable analysis, dual triggering was not associated with mature oocyte yield, while age and AMH remained significant predictors (p < 0.001).

**Conclusion:**

Dual triggering does not improve mature oocyte yield or clinical outcomes in POSEIDON group 4 patients, supporting no clear benefit for its routine use in this population.

## Introduction

Poor ovarian responders (PORs) represent a significant subset of patients undergoing assisted reproductive technology (ART) and remain one of the most challenging populations in reproductive medicine. In this context, where the reproductive window is exceedingly narrow, maximizing the number of mature oocytes retrieved is critical, as even modest increases in oocyte yield can meaningfully affect reproductive outcomes. A systematic review by Oudendijk et al. in poor responders showed pregnancy rates of 0–7% when only one oocyte was retrieved, compared with 11.5–18.6% when four oocytes were obtained (1).

Final oocyte maturation triggering represents a crucial step in controlled ovarian stimulation. Human chorionic gonadotropin (hCG) has traditionally been used as the sole agent to induce ovulation due to its structural similarity to luteinizing hormone (LH) (2).

On the other hand, GnRH agonists (GnRHa) promote the release of not only LH but also FSH, which synergizes with LH by enhancing granulosa cell LH receptor expression and driving key peri-ovulatory processes (3, 4).

GnRH agonists (GnRHa) induce an endogenous surge of both LH and FSH. The LH surge activates granulosa cell-mediated peri-ovulatory signaling, including the release of EGF-like peptides, which promote cumulus expansion, meiotic resumption, and the coordinated maturation of the cumulus–oocyte complex. The accompanying FSH surge may further support granulosa cell function and cumulus expansion (5).

The term dual trigger refers to the combination of GnRHa and hCG for final oocyte maturation. This approach has shown promise in improving oocyte maturity and reproductive outcomes in normo-responders (6, 7).

However, evidence for this approach in PORs remains limited and conflicting, primarily due to the marked heterogeneity within this population. Indeed, despite considerable efforts, a universally accepted definition of poor ovarian response is still lacking. The Bologna criteria, introduced by ESHRE in 2011, represented an important attempt at standardization but encompass highly heterogeneous phenotypes, a limitation that may bias comparative analyses and mask subgroup-specific responses to different treatment strategies (8, 9).

To overcome these limitations, the POSEIDON classification was developed to distinguish expected (groups 3 and 4) from unexpected poor responders (groups 1 and 2) based on age, ovarian reserve biomarkers, and prior stimulation outcomes, offering a more prognostically relevant stratification (10).

Among these, POSEIDON group 4, comprising patients who are ≥35 years old, with AFC <5 and/or AMH <1.2 ng/mL, represents a particularly challenging phenotype, characterized by advanced maternal age and reduced ovarian reserve, thus combining both quantitative and qualitative impairment of oocyte competence.

The clinical relevance of this subgroup is further amplified by the current trend toward delayed childbearing (11), with women over 40 years now accounting for approximately 18% of IVF cycles in Europe (12). To date, data specifically addressing the role of dual triggering in POSEIDON group 4 patients remain scarce (13).

The objective of the present study was therefore to evaluate whether dual triggering improves the number of mature oocytes retrieved compared with hCG alone in POSEIDON group 4 patients.

## Methods

### Study design and population

This retrospective cohort study included women undergoing *in vitro* fertilization (IVF) cycles who fulfilled the criteria for POSEIDON group 4, defined as age ≥35 years with an antral follicle count (AFC) <5 and/or anti-Müllerian hormone (AMH) <1.2 ng/mL. All eligible cycles were performed at a tertiary-level assisted reproduction center (EmbryoLab Fertility Clinic, Thessaloniki, Greece) between 1 January 2018 and 1 June 2024.

The study was approved by the local ethics committee (Approval No. 17/07/2024).

All women undergoing intracytoplasmic sperm injection (ICSI) within a GnRH antagonist ovarian stimulation protocol were eligible for inclusion. Cycles involving conventional IVF, preimplantation genetic testing, oocyte donation, or fertility preservation were excluded. In addition, cycles undergoing an elective freeze-all strategy were excluded from the analysis.

Patients presenting with uterine anomalies or endocrine disorders were also excluded from the analysis.

### Ovarian stimulation and ART procedures

Controlled ovarian stimulation was initiated on menstrual cycle day 2 or 3 following exclusion of ovarian cysts or endometrial abnormalities by transvaginal ultrasound. The starting dose of gonadotropins (rFSH: Gonal-f -Merck Serono, Geneva, Switzerland- e/o Puregon -Organon/MSD, Oss, The Netherlands-hMG: Menopur -Ferring Pharmaceuticals, Saint-Prex, Switzerland) ranged from 150 to 450 IU per day and was individualized according to patient age, body mass index (BMI), ovarian reserve markers, and previous response to stimulation. Follicular development was monitored by serial transvaginal ultrasound beginning on stimulation day 5 or 6. A GnRH antagonist (Cetrotide -Merck Serono, Geneva, Switzerland) was introduced when at least one follicle reached a mean diameter of ≥12 mm in order to prevent a premature luteinizing hormone (LH) surge. Final oocyte maturation was triggered when at least one follicle reached a mean diameter of ≥17 mm.

In the dual-trigger group, final oocyte maturation was induced using a combination of a GnRH agonist (triptorelin acetate 0.2 mg- Decapeptyl -Ipsen) and recombinant human chorionic gonadotropin (rhCG 250 µg,Ovitrelle -Merck Serono). In the control group, ovulation triggering was performed using rhCG alone.

The choice between dual trigger and hCG-only trigger was made at the discretion of the treating physician according to routine clinical practice, as no standardized institutional protocol for trigger selection was in place during the study period.

Transvaginal ultrasound-guided oocyte retrieval was scheduled 35–37 hours after triggering in all cases. Intracytoplasmic sperm injection was performed in all cycles according to standard laboratory protocols. Luteal phase support consisted of vaginal progesterone (Utrogestan, Besins Healthcare, Chatswood, Australia – Vasclor, Verifield, London, UK) initiated on the day of oocyte retrieval and continued for at least six weeks or until a negative pregnancy test.

### Outcomes measures

The primary outcome was the number of mature oocytes retrieved per cycle, defined as the absolute number of metaphase II (MII) oocytes obtained at oocyte retrieval. Secondary outcomes included the total number of oocytes retrieved, oocyte maturation rate, fertilization rate, defined as the proportion of normally fertilized two-pronuclear (2PN) embryos relative to the number of injected MII oocytes, and embryo utilization rate, defined as the proportion of embryos either transferred or cryopreserved relative to the total number of 2PN embryos obtained. Clinical outcomes were evaluated per initiated cycle. Clinical pregnancy was defined as a pregnancy diagnosed by ultrasonographic visualization of one or more gestational sacs. Miscarriage was defined as pregnancy loss before 22 weeks of gestation, and live birth was defined as the delivery of at least one live-born infant beyond 24 weeks of gestation (14).

### Statistical analysis

All analyses were conducted at the cycle level. Continuous variables are presented as mean ± standard deviation and were compared using Student’s t-test or Mann–Whitney U test, as appropriate. Categorical variables are presented as counts and percentages and were compared using the χ^2^ test or Fisher’s exact test. To account for within-patient correlation due to multiple cycles per woman, generalized estimating equation (GEE) regression models were used to evaluate the independent association between triggering strategy and the primary outcome (number of MII oocytes retrieved). Covariates included in the multivariable model were age, AMH, BMI, total gonadotropin dose, and the number of previous failed attempts. To account for potential temporal changes in clinical and laboratory practice, treatment cycles were additionally categorized into three calendar periods and included in the adjusted GEE model.

No missing data were present for the variables included in the final analyses; therefore, no imputation procedures were performed.

Results were reported as regression coefficients (β) with 95% confidence intervals (CI). The assumptions for the final model were successfully tested. All statistical tests used a two-tailed α of 0.05. Analyses were performed using STATA 13.0. A p-value < 0.05 was considered statistically significant.

## Results

A total of 1,824 IVF cycles in women classified as POSEIDON group 4 were included in the final analysis. Of these, 1,470 cycles were triggered with recombinant human chorionic gonadotropin (rhCG) alone, and 354 cycles received a dual trigger.

Baseline characteristics were largely comparable between groups (**Table 1**).

**Table 1:** Baseline characteristics of POSEIDON group 4 cycles according to triggering strategy.

Variable	hCG only (n = 1470)	Dual trigger (n = 354)	P value
Age (years)	39.27 ± 2.37	39.61 ± 2.45	0.02
Body mass index (kg/m^2^)	24.96 ± 4.72	25.07 ± 4.55	0.46
AMH (ng/mL)	0.636 ± 0.311	0.637 ± 0.307	0.97
Previous ART cycles (n)	1.74 ± 2.03	1.71 ± 1.73	0.36

Data are presented as mean ± standard deviation (SD). P values were calculated using the Mann–Whitney U test.

Mean age was slightly higher in the dual-trigger group compared with the rhCG-only group (39.6 ± 2.4 vs 39.3 ± 2.4 years, p = 0.02), while body mass index did not differ significantly (25.1 ± 4.6 vs 25.0 ± 4.7 kg/m^2^, p = 0.46). Markers of ovarian reserve were similar, with no significant difference in AMH levels (0.64 ± 0.31 vs 0.64 ± 0.31 ng/mL, p = 0.97). The number of previous ART cycles was also comparable between groups (1.71 ± 1.73 vs 1.74 ± 2.03, p = 0.36).

Ovarian stimulation parameters, laboratory outcomes, and clinical results according to triggering strategy are summarized in **Table 2**.

**Table 2:** Ovarian stimulation, laboratory and clinical outcomes according to triggering strategy in POSEIDON group 4 cycles.

Variable	hCG only (n = 1470)	Dual trigger (n = 354)	P value
Ovarian stimulation characteristics			
Stimulation duration (days)	10.45 ± 2.11	10.19 ± 2.10	0.051
Total gonadotropin dose (IU)	2318 ± 755	2378 ± 717	0.091
Oocyte yield and maturation			
Oocytes retrieved (n)	4.61 ± 3.10	4.99 ± 3.59	0.25
Mature (MII) oocytes (n)	3.79 ± 2.72	4.02 ± 3.05	0.43
Oocyte maturation rate (%)	82.69 ± 23.40	80.92 ± 25.07	0.32
Embryology outcomes			
Fertilization rate (%)	69.87 ± 30.90	74.15 ± 29.85	0.04
Embryo utilization rate (%)	70.37 ± 34.18	62.41 ± 35.02	<0.001
Cryopreserved embryos per cycle (n)	0.59 ± 1.11	0.59 ± 1.00	0.60
Embryos transferred per cycle (n)	1.10 ± 0.75	1.00 ± 0.56	—
Complete embryo attrition, n (%)	1000 (68.0)	244 (68.9)	0.76
Embryo transfer strategy			
Day 3 transfer	967/1176 (82.2%)	216/301 (71.8%)	<0.001
Day 5 transfer	209/1176 (17.8%)	85/301 (28.2%)	
Clinical outcomes per initiated cycle			
Clinical pregnancy	367/1470 (25.0%)	81/354 (22.9%)	0.413
Miscarriage	164/367 (44.7%)	36/81 (44.4%)	0.531
Live birth	203/1470 (13.8%)	45/354 (12.7%)	0.589

Data are presented as mean ± standard deviation (SD) or n/N (%). Continuous variables were compared using the Mann–Whitney U test and categorical variables using the χ^2^ test. Transfer-stage analyses were performed only among cycles reaching embryo transfer.

The duration of ovarian stimulation was slightly shorter in the dual-trigger group (10.2 ± 2.1 vs 10.4 ± 2.1 days, p = 0.051), whereas total gonadotropin consumption did not differ significantly (2,378 ± 717 vs 2,318 ± 755 IU, p = 0.09).

### Laboratory and clinical outcomes

The total number of oocytes retrieved was comparable between groups (5.0 ± 3.6 vs 4.6 ± 3.1, p = 0.25), as was the number of mature (MII) oocytes (4.0 ± 3.0 vs 3.8 ± 2.7, p = 0.43), resulting in similar oocyte maturation rates (80.9 ± 25.1% vs 82.7 ± 23.4%, p = 0.32). Fertilization rates were slightly higher in the dual-trigger group (74.1% vs 69.8%; p = 0.04), corresponding to an absolute difference of 4.3 percentage points (95% CI 0.6–8.0). Although this did not translate into improved downstream outcomes. Indeed, the embryo utilization rate was lower with dual triggering (62.4 ± 35.0% vs 70.4 ± 34.2%, p < 0.001), while the mean number of cryopreserved embryos per cycle remained similar (0.59 ± 1.00 vs 0.59 ± 1.11, p = 0.60).

No significant difference was observed in the rate of complete embryo attrition, defined as no embryo available for transfer or cryopreservation, between the hCG-only and dual-trigger groups (68.0% vs 68.9%, p = 0.76).

A higher proportion of blastocyst-stage transfers was observed in the dual-trigger group (28.2% vs 17.8%), whereas cleavage-stage transfers were more frequent in the rhCG-only group (82.2% vs 71.8%) (χ^2^ p < 0.001). Despite these differences in transfer strategy, clinical outcomes were comparable between groups, with similar clinical pregnancy rates per initiated cycle (22.9% vs 25.0%, p = 0.41), miscarriage rates (44.7% vs 44.4%, p = 0.53), and live birth rates (12.7% vs 13.8%, p = 0.59).

### Multivariable analysis

Generalized estimating equation (GEE) regression analysis accounting for repeated cycles per patient confirmed that dual triggering was not independently associated with the number of mature oocytes retrieved (β = 0.29, 95% CI −0.03 to 0.60, p = 0.074).

In contrast, chronological age was a significant negative predictor of MII yield (β = −0.15, 95% CI −0.20 to −0.09, p < 0.001), while AMH was strongly and positively associated with the number of mature oocytes (β = 3.26, 95% CI 2.83 to 3.68, p < 0.001). BMI and total gonadotropin dose were not independently associated with MII yield (p = 0.25 and p = 0.13, respectively).

The inclusion of the number of previous failed attempts in the adjusted model did not change the results and was not independently associated with MII oocyte yield (β = −0.02, 95% CI −0.08 to 0.05, p = 0.637).

Adjustment for calendar period did not materially change the effect estimates observed in the primary analysis (**Table 3**).

**Table 3:** Multivariable GEE analysis with number of MII oocytes as the dependent variable.

Variable	β (95% CI)	P value
Dual trigger (vs hCG)	0.29 (−0.03 to 0.60)	0.074
Age (years)	−0.15 (−0.20 to −0.09)	<0.001
Body mass index (kg/m^2^)	−0.02 (−0.05 to 0.01)	0.246
AMH (ng/mL)	3.26 (2.83 to 3.69)	<0.001
Total gonadotropin dose (IU)	0.00013 (−0.00004 to 0.00029)	0.129
Previous failed attempts	−0.02 (−0.08 to 0.05)	0.637
Calendar year	0.06 (−0.14 to 0.26)	0.549

GEE, generalized estimating equations; MII, metaphase II; AMH, anti-Müllerian hormone; hCG, human chorionic gonadotropin.

## Discussion

In this study of women classified as POSEIDON group 4, the use of dual triggering with a GnRH agonist plus hCG did not lead to an increased number of mature (MII) oocytes compared to hCG alone. This lack of benefit was evident both in unadjusted comparisons and after adjustment for relevant confounders. Moreover, dual triggering did not translate into improvements in clinical outcomes, such as clinical pregnancy or live birth rates. Although fertilization rates were slightly higher in the dual-trigger group, the absolute difference was modest (4.3 percentage points). Moreover, this finding was no longer statistically significant after adjustment for multiple comparisons and was not associated with improvements in downstream reproductive outcomes, including pregnancy and live birth rates. Therefore, its clinical relevance remains uncertain.

Conversely, the embryo utilization rate was lower in the dual-trigger group despite a higher proportion of blastocyst transfers. One possible explanation is the greater use of extended embryo culture in this group, which may have increased embryo attrition, particularly in this low-prognosis population. However, detailed data regarding blastocyst development and culture outcomes were not consistently available, and therefore no definitive conclusions can be drawn regarding the mechanisms underlying this observation.

Ovulation triggering represents a critical step in IVF, and in selected clinical scenarios, tailoring the triggering approach to patient characteristics may influence cycle performance (15). However, our results suggest that such an approach may have limited utility in POSEIDON group 4 patients.

POSEIDON group 4 represents a particularly severe poor-prognosis phenotype, combining advanced maternal age with reduced ovarian reserve, thereby integrating both quantitative limitations (low oocyte yield) and qualitative constraints (age-related decline in oocyte competence). Under these biological conditions, optimization of final outcomes over the last 36 hours—although mechanistically appealing—may have limited ability to overcome age-related impairment in meiotic competence and embryo developmental potential. Consistent with this, in our adjusted analysis, chronological age was independently and negatively associated with MII yield, whereas AMH was strongly and positively associated with MII number; in contrast, the triggering strategy was not independently associated with the primary endpoint. Taken together, these results support the concept that in POSEIDON group 4, biological constraints may outweigh pharmacological optimization of triggering. Although the addition of a GnRH agonist may optimize the periovulatory endocrine environment and the processes leading to nuclear maturation, the acquisition of developmental competence is a progressive process that extends throughout folliculogenesis. Experimental and translational studies have demonstrated the importance of cumulus–oocyte communication and the accumulation of maternal transcripts and other cellular machinery required to support subsequent embryo development. Accordingly, an oocyte reaching the MII stage may not necessarily possess full cytoplasmic and developmental competence (5, 16).In women with POSEIDON group 4 characteristics, modification of the final maturation signal may have limited capacity to overcome constraints established during earlier stages of follicular development. Although cytoplasmic competence and the molecular mechanisms underlying oocyte quality were not directly assessed in the present study, our findings are consistent with the concept that optimization of nuclear maturation alone may not be sufficient to improve downstream reproductive outcomes in this population.

It is well established that the population of older patients largely overlaps with that of low-prognosis patients. In this subgroup, available data remain highly heterogeneous, and a clear consensus appears even further from being reached.

Although a statistically significant age difference was observed between groups, the absolute difference was small (0.34 years), and age was accounted for in the multivariable analysis.

Results across studies remain inconsistent, likely due to the lack of a universally accepted definition of poor ovarian responders and the resulting heterogeneity of studied populations, even when applying the Bologna criteria. As highlighted by Papathanasiou (17), these criteria encompass multiple distinct clinical phenotypes based on age, ovarian reserve, and prior response. Aggregating such heterogeneous profiles under a single category may introduce bias, limit external validity, and mask differential responses to treatment across subgroups (**Figure 1**).

**Figure 1:**
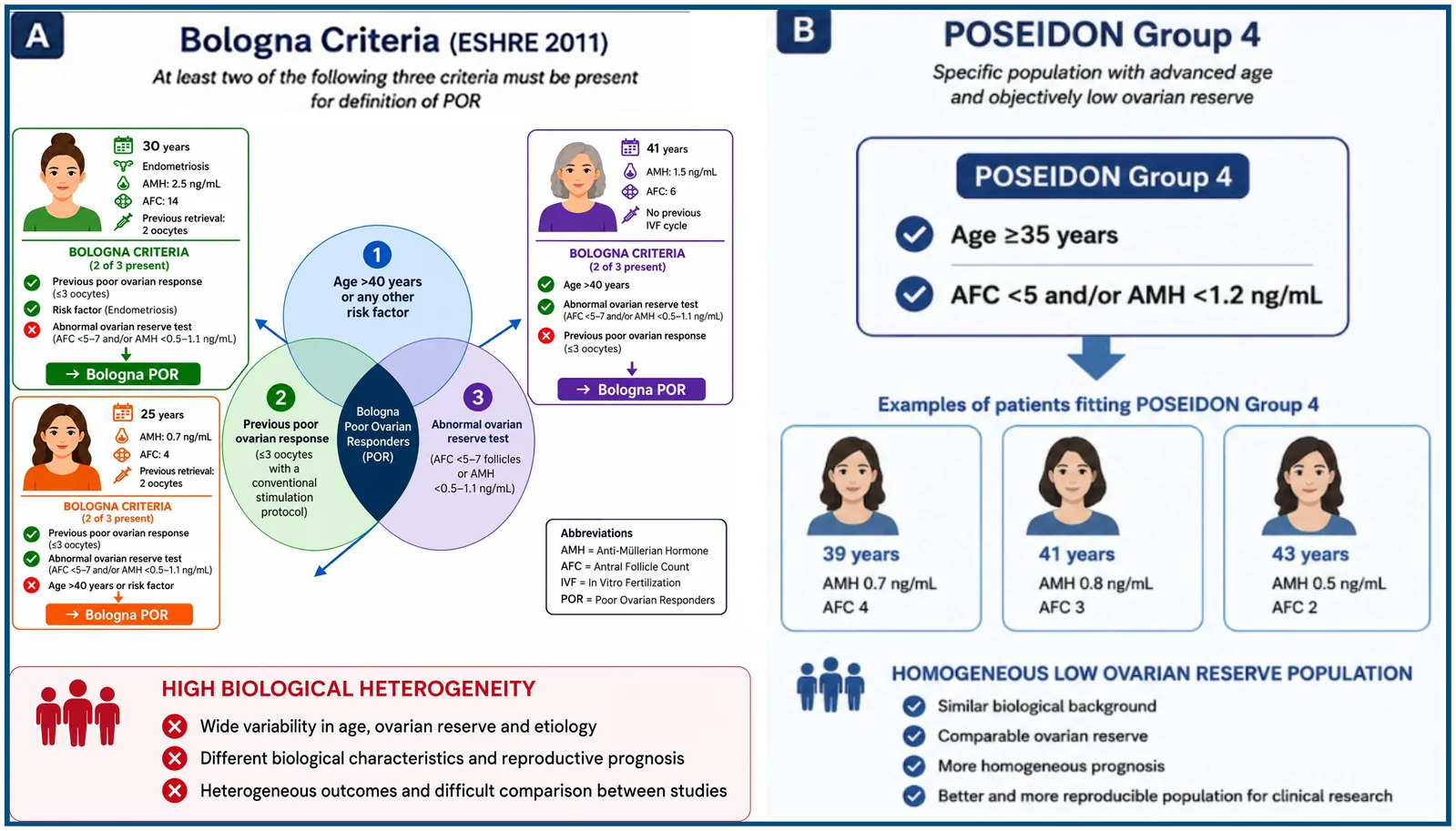
Clinical heterogeneity of bologna poor ovarian responders and characteristics of POSEIDON group 4. **(A)** Distinct clinical phenotypes fulfilling the Bologna criteria for poor ovarian response. **(B)** Definition and key characteristics of women classified as POSEIDON Group 4.

A meta-analysis comparing dual versus single triggering in poor ovarian responders reported higher clinical pregnancy and live birth rates with dual triggering; however, the included studies were characterized by substantial heterogeneity in both case definition and patient age, encompassing mixed populations defined by the Bologna criteria as well as POSEIDON groups 3 and 4. Moreover, this meta-analysis primarily focused on downstream clinical endpoints, such as clinical pregnancy, implantation, and live birth rates, without systematically addressing biologically proximal outcomes directly related to ovarian response, such as the number of mature (MII) oocytes (18).

This heterogeneity in case definition, together with the reliance on clinical outcomes that are susceptible to confounding—such as male factor infertility and maternal characteristics not directly related to ovarian function—limits the interpretability of these findings and their applicability to specific low-prognosis patient subgroups.

In contrast, a recent meta-analysis focusing on proximal outcomes, including oocyte yield and MII number, reported less consistent results, indicating that the benefit of dual triggering is not uniform across phenotypes and suggesting a more nuanced and less definitive effect (13).

Evidence comparing dual versus single triggering specifically in POSEIDON group 4 patients remains limited. Two older retrospective studies (Chern et al. (19) and Tulek et al. (20)) reported more favorable outcomes with dual triggering, in contrast with our findings. However, both analyses differ in methods and analysis, without accounting for within-patient variability across repeated cycles.

In the study by Chern et al., chronological bias represents a major concern: dual triggering was predominantly adopted after 2015, whereas hCG-only triggering was mainly used in earlier periods. This raises the possibility that improvements over time in stimulation protocols, laboratory systems, vitrification practices, and embryo transfer policies may have confounded the observed differences, potentially attributing to the triggering strategy effects that partly reflect the natural evolution of IVF practice. Finally, the relatively small sample size of this study (308 cycles in total) further limits the robustness and generalizability of the reported findings.

Similar methodological concerns apply to the retrospective study by Tulek et al., where ovarian reserve characterization was limited, with anti-Müllerian hormone (AMH) levels not reported despite the classification of patients as POSEIDON group 4.

Moreover, outcomes were analyzed at the patient level rather than at the cycle level, without accounting for within-patient variability across repeated cycles using appropriate methods such as GEE modeling. This analytical approach may obscure cycle-specific effects and variability in ovarian response over successive attempts.

The present study offers several strengths. First, it includes a large number of treatment cycles, enhancing statistical power in a population that is often underrepresented in randomized trials. Second, the analysis focuses on a strictly defined prognostic subgroup, POSEIDON group 4, thereby reducing clinical heterogeneity and improving interpretability. Third, the primary outcome was the number of mature (MII) oocytes retrieved, a biologically proximal and clinically relevant endpoint that directly reflects ovarian response and is less susceptible to confounding by laboratory practices or male factor variability than downstream outcomes such as clinical pregnancy or live birth. Finally, outcomes were analyzed at the cycle level while appropriately accounting for within-patient correlation across repeated cycles through the use of generalized estimating equation regression models, a methodological approach that is frequently overlooked in retrospective IVF studies.

Nevertheless, some limitations must be acknowledged. The retrospective design inherently carries a risk of residual confounding and indication bias, despite adjustment for multiple relevant covariates. In particular, the choice of triggering strategy was left to the treating physician’s discretion and was therefore susceptible to selection bias and confounding by indication.

Information regarding parameters such as follicular size distribution on the day of triggering and antral follicle count were not consistently recorded in the electronic database and were therefore unavailable for retrospective analysis. In addition, variations in center-specific laboratory protocols and clinical practices may have influenced selected intermediate outcomes, potentially limiting the external generalizability of the findings.

## Conclusion

Our findings show that dual triggering offers no meaningful advantage over hCG alone in POSEIDON group 4 women. The biological limitations of advanced age and low ovarian reserve may limit the effectiveness of such strategies. Prospective studies specifically designed to address POSEIDON group 4 patients are warranted to confirm these findings and to identify interventions capable of meaningfully improving outcomes in this particularly challenging population.

## Data Availability

The raw data supporting the conclusions of this article will be made available by the authors, without undue reservation.
